# Deflationism, explanation and “because”

**DOI:** 10.1007/s11098-025-02351-7

**Published:** 2025-06-03

**Authors:** Julio De Rizzo

**Affiliations:** Department of Philosophy, University of Vienna, Vienna, Austria

**Keywords:** Truth, Deflationism, Disquotationalism, Ground, Explanation, ‘Because’

## Abstract

Two influential objections to deflationism about truth question its ability to explain the role of true beliefs in successful actions; and to account for general compositional principles linking truth to complex sentences governed by truth-functional connectives. In this paper, I address recent formulations of these objections by Will Gamester and Richard Heck. My responses draw on recent work on explanation, grounding, and the logic of “because”. However, each response leaves a residual concern for deflationists that these strategies alone cannot fully resolve. In the final section, I propose a view I call "Aristotelian Deflationism," which incorporates specific “because” principles relating to truth as an alternative to standard instances of the T-schema. While not deflationist in the strictest sense, I argue that this approach offers compelling ways to address both the primary objections and the residual concerns effectively.

## Introduction

A prominent form of deflationism holds that the meaning of the truth predicate is fully exhausted by the instances of the T-schema (cf. Wright, 1992: 13; Horwich, 1998: 36–38)^1^:

1. “p” is true if and only if p.

As Field succinctly puts it, “truth is at bottom disquotational” (Field, 1994: 222; Quine, 1970: 12). On the positive side, the disquotationalist view asserts that truth serves certain expressive purposes. For example, it allows us to express generalizations that would otherwise be inexpressible, such as, “Every axiom of Peano Arithmetic is true” (Quine, op. cit.; Halbach, 1999). It also enables indeterminate discourse about someone’s words, thoughts, or beliefs, as in “What Elisa said is true” (Horwich, 1998: 3).^2^ The same uses apply to falsity as well.

On the negative side, disquotationalism significantly limits the role of truth: truth is not meant to serve any distinctive explanatory function. In other words, truth-talk cannot enable us to explain phenomena that would otherwise be unexplained.^3^

One of the most frequently discussed criticisms of deflationism challenges this restriction. Opponents argue, through the so-called “Success Argument,” that typical explanations of successful actions rely on truth in ways that go beyond its supposed purely disquotational nature. We will refer to this challenge as “the success objection.”

Alongside the sucess objection, some opponents of deflationism urged that it fails on broadly logical grounds (Heck, 2018, 2021; Ketland, 1999; Shapiro, 1998). More specifically, they argue that the truth predicate disquotationally characterized cannot do justice to compositional principles for logical connectives, for instance^4^:

2. “p & q” is true iff “p” is true and “q” is true.

These principles arguably constitute our basic understanding of truth and its relation to logical connectives, and a theory of truth loses much of its significance and utility without them (Tarski, 1935: 257). We call this “the logical objection.”

In framing the logical objection, the expression “cannot do justice” is intentionally left vague for the purposes of generality. In classical logic, instances of the T-schema alone do not entail the corresponding instances of the compositional principles. Even the universal closure of the T-schema—obtained, for instance, by quantifying substitutionally into sentence position—fails to imply the universal closure of such compositional principles.^5^

A widely discussed response is to include instances of the compositional principles in the characterization of truth (e.g. Lepore & Ludwig, 2007: 31–32). This move, however, raises a crucial question: does this addition exceed the boundaries set by deflationist principles? Should deflationists avoid this addition given their minimalist commitments? A related concern, especially relevant when extending from formal to natural languages, is how to determine where to limit the inclusion of compositional principles once they are allowed in the characterization of truth (cf. Heck, 2021; see below).

Both the success and logical objections, in various forms, have sparked enduring debates between deflationists and their critics. In this paper, I address two relatively recent iterations of these objections: Gamester’s (2018) version of the success objection and Heck’s (2021) version of the logical objection, which is framed as a response to Field (2006). I examine each on behalf of the deflationist in Sects. 2 and 3, respectively. While the success and logical objections are largely distinct, in the forms considered here both center on issues of explanation and rely on the connective “because” to express explanations involving truth-ascriptions. This shared conceptual framework enables us to engage with a common body of literature on explanatory reasoning and the logic of “because,” allowing us to identify underlying similarities and to develop a more unified, comprehensive response.

The replies I offer in Sects. 2 and 3 adhere to the deflationist's minimalist constraints. However, with each objection, a residual concern remains, manifesting as a concession to the objector. Addressing these concerns points toward an account of truth that incorporates the connective “because” within instances of the T-schema, a view I label “Aristotelian Deflationism.” In Sect. 4, I explore the advantages of this account in light of the preceding discussion. I argue that, while it may not align with strict orthodox deflationism, this approach has significant merits worth considering.

## Truth, explanation and success

True beliefs facilitate and help explain successful actions. If Elisa wants a beer and believes that nodding will get her one, then—assuming no other belief or intention overrides this one, and barring any physical limitations (assumptions that I leave implicit hereafter)—Elisa will nod. If her belief is true, she achieves her goal; crucially, *because* her belief is true, her action successfully fulfills her desire. The non-deflationist objection, often framed as the “Success Argument,” contends that deflationism fails to adequately capture the explanatory role that truth plays in such everyday explanations of successful behavior (Putnam, 1978, Field 1986, Leeds, 1995, Kitcher, 2002).

To clarify this objection, let us first distinguish two relevant senses of “explanation” (Bromberger, 1966). In the *act sense*, an explanation is a kind of speech act, conducted by an agent for specific purposes, with certain success conditions, duration, and linguistic context. In the *content sense*, an explanation refers to what is expressed by some statements, especially those containing terms like “because,” “in virtue of,” and “explains why.” In what follows, I focus on explanation in the content sense.

Not all uses of “because” are explanatory in the relevant sense. For instance, in “The Smiths are home because their lights are on,” the clause following “because” does not plausibly state *a reason why* the Smiths are home. Rather, it offers a reason to *believe* they are home—an example of the so-called evidential use of “because” (Morreall, 1979; Bolzano, 1837: Sect. 198). This contrasts with the non-evidential, or objective use, illustrated by: “The Smiths are home because they are expecting a delivery.” Here, the sentence does offer an explanation—in this case, a cause—for why they are home. In what follows, I focus exclusively on the objective use of “because.”

In typical cases like the one described, at least two explanations are in play: one explaining the action itself—Elisa’s nodding—and another explaining the success of that action relative to her goal, that is, the satisfaction of her desire. These explanations correspond to two distinct “why” questions:

1. Why did Elisa nod?
2. Why was Elisa’s nodding successful?

For present purposes, we can largely set aside the exact form of the first explanation. We adopt a widely accepted approach: that beliefs and desires explain actions. Thus, in response to the first question, we might say that Elisa nodded because she believed that nodding would get her a beer, and she wanted a beer. We need not resolve whether “because” here denotes a causal relationship or some other explanatory connection, such as motivation or a sui generis form of explanation (see e.g. Mele, 2013). I refer to such explanations as *action explanations*. Notably, the truth of the belief is not at issue in action explanations. Whether true or false, the belief can lead to an action either way.

Matters change once we turn to the second explanation, i.e. the answer to the second question. For deflationists and their opponents agree that actions which succeed in bringing about a desired output not only strongly correlate with but are (at least partially) *explained by* true beliefs about them. Henceforth call these *success explanations*.

How does a success explanation look like? Notice, first, that our explanandum, what we want explained, is not the mere output of the action, e.g. that Elisa got a beer. For that leaves out the important feature that she *desired it to happen*, and furthermore *targeted that outcome with her action*. This motivates the specific form the second question takes: instead of asking why did Elisa get a beer, what we seek an answer to is: why did Elisa satisfy her desire for a beer; or: why was her nodding successful relatively to that desire? The following seems a correct answer:

Elisa satisfied her desire for a beer *because* (she nodded *and* by nodding, she got a beer).

That is, the action plays a part in satisfying the desire, together with the fact that the action brought about the desired outcome.

From the two explanations we have so far, i.e.:

(*Action explanation*): Elisa nodded *because* Elisa believed that by nodding she would get a beer & she wanted a beer

And.

(*Success explanation*): Elisa satisfied her desire for a beer *because* Elisa nodded & her nodding caused her to get a beer.

And a form of transitivity for “because”,^6^ we obtain:

3. Elisa satisfied her desire for a beer *because* (Elisa believed that by nodding she would get a beer & by nodding, she got a beer).

In (3), Elisa’s belief about the means that lead her to the end of satisfying her desire gets assigned an explanatory role. One could accept statement (3) independently of the preceding derivation. However, the derivation reveals that accepting certain plausible principles regarding “because” and action explanations *commits* one to (3). Specifically, assuming the *Action Explanation*, *Success Explanation*, and the transitivity of “because”, deflationists and non-deflationists alike should find (3) uncontroversial.

The core of the debate centers on the relationship between (3) and the following possible explanation:

4. Elisa satisfied her desire for a beer *because* Elisa believed *truly* that by nodding she would get a beer.

The key issue is whether explanations like (3) fall short in comparison to those like (4). In other words, do explanations that involve true belief, such as (4), provide more than what is expressed by explanations that omit reference to truth, as in (3)? If they do, this supports the non-deflationist claim that deflationism cannot fully capture the role of truth in everyday explanations of successful actions. There are two main ways the non-deflationist point can be established:

1. *Differing Extensions*: If explanations of the forms exemplified by (3) and (4) are not always alike in truth-value, then explanations referencing truth (like in (4)) would indeed capture a distinct, broader explanatory domain than explanations like (3).
2. *Explanatory Strength*: If (4) provides a *stronger* or in some broad sense *better* explanation of the success of actions than (3), then (4) ought to be preferred over (3).

### The success objection: Gamester’s problem case

Recently, Gamester (2018) pursued the second strategy: according to him, the deflationist’s explanation of sucess actions without truth, along the lines of (3), misses important explanatory features of some interesting cases.

Before presenting the case that is meant to spell trouble for the deflationist, Gamester illustrates the deflationist’s strategy with a simple case. The examples describe situations of participants in the show “Be a Millionaire!”, in which the contestant is asked to choose among two opaque boxes X and Y in front of them, one of which contains a cheque with a million pounds placed by the host. If they choose the right box, they get the money. We suppose that contestants want to win the money. The simple case is:

*Alison*. The host puts the cheque in box X. Alison chooses box X. She wins the money. (Gamester, 2018: 1245)

Alison’s action of choosing the box is thus successful with respect to the desire of winning the money. We proceed as before. First, Alison’s action might be explained along the following lines:

(*Action explanation*): Alison chose box X because (she believed that by choosing it, she would get the money; and she wanted the money).

The success of Alison’s action, in turn, is explained thus:

(*Sucess explanation 1*): Alison satisfied her desire to get the money because (she chose box X; and by choosing X, she got the money).

Again with the aid of transitivity of “because”, we get

(*Success explanation 2*): Alison satisfied her desire to get the money because (she believed that by choosing it, she would get the money; and by choosing X, she got the money).

Which is intimately related to the non-deflationist’s explanation, namely

Alison satisfied her desire to get the money because *she truly believed* that by choosing it, she would get the money.

Notice that Alison’s belief about the money’s location—that it was in box X—should be distinguished from her belief that choosing box X would result in obtaining the money. Plausibly, the former supports the latter: because she believed the money was in box X, she also believed that selecting it would secure the money.

Now to the problem case:

*Callum*. The host puts the cheque in box X. Unbeknownst to Callum, the cheque is surreptitiously moved to box Y. Callum chooses box X. In the audience is an eccentric billionaire, who loves box X. She is so pleased that Callum has chosen her favourite box that she gifts him a million pounds. (Gamester, 2018: 1251)

Though Callum does not win the game, Callum’s action is succesful with respect to the desire of getting the money, or becoming a millionaire (as was Alison with respect to those same desires). But unlike Alison, his belief with respect to the alocation of the cheque—that it is in box X, which also prompts his belief that by choosing X, he would get the money, therefore also his action—is *false*.

As Gamester notes, Callum’s case ought to be distinguished from a case where the action is simply unsuccessful, say, if one chooses the wrong box and ends up with no money (see Bryan’s case in 2018: 1245). But the most important contrast in this context is between Alison and Callum, for

(…) there is a salient difference between Callum’s success and Alison’s success. While Alison’s success is to be expected, Callum’s is quite out of the blue—the fact that the action he *thought* would result in the satisfaction of his desire *in fact* turned out to do so is a *sheer coincidence*. (Gamester, 2018: 1251; italics original)

However, the very same strategy that the deflationist uses for Alison case hold mutatis mutandis to Callum’s case as well:

(Action explanation*): Callum chose the box X because (he believed that by choosing it, he would get the money; and he wanted the money).

(*Sucess explanation* 1*): Callum satisfied his desire to get the money because (he chose box X; and by choosing X, he got the money).

(*Success explanation* 2*): Callum satisfied his desire to get the money because (he believed that by choosing it, he would get the money; and by choosing X, he got the money).

That is, as far as these explanations go, deflationists treat Alison’s and Callum’s cases on a par. Gamester writes:

However, if the deflationist is right that all there is to the explanation of Alison’s success is [Action Explanation, Success Explanation 1, Success explanation 2], just as all there is to the explanation of Callum’s success is [Action Explanation*, Success Explanation 1*, Success explanation 2*] then there is no such contrast: the two are on an explanatory footing. If this is right, then we lose all right to say that there is any non-/coincidental contrast between the cases; and this is a straightforwardly disastrous result. To treat every instance of practical success—coincidental and non-coincidental—in this explanatorily identical way is to treat the entire phenomenon as coincidental and hence mysterious. (1254–1255)^7^

The deflationist’s strategy thus cannot do justice to the important, explanatory distinction between coincidental and non-coincidental successes. Crucially, according to Gamester, the non-deflationist’s (the “inflationist” in his words) candidate is superior in this respect:

(…) it is simply obvious what the inflationist will say at this point. The inflationist will appeal to some explanatory premise that invokes truth: that actions that result from true beliefs tend to be successful (i.e. effectively pertinent with regards to the desires that they result from), for example. Since the beliefs that Alison’s action resulted from are true, this explains the success of Alison’s action; and since the beliefs that Callum’s action resulted from are not true, it cannot do so for Callum. (1255; see also 1258.)

Thus, that Alison’s beliefs were all true—including the one about the money’s location—drives a wedge between the cases, and thus allegedly captures the targeted distinction between coincidental and non-coincidental successes.

### A response

My response consists of two related points. First, a relatively minor one: as Gamester carefully notes, the Success objection poses a problem only if the deflationist is committed to asserting that *all there is* to Alison’s and Callum’s successes is captured by those explanatory claims alone. These we may take to include the belief about the location of the money, that plausibly prompts the choices of both Alison and Callum. Since Alison’s case involves true beliefs driving actions with successful outcomes, the deflationist does appear committed to this approach for her case. However, since in Callum’s case the belief about the money’s location is false, why should the deflationist be confined to such claims? I see no compelling reason for this restriction. It may be that every supplementary explanation that goes beyond such claims ultimately fails to capture the coincidence/non-coincidence distinction (as Gamester suggests: 1256–1261), but I will argue shortly that this is incorrect. Up to now, deflationists have provided ways of addressing cases like Alison’s, where non-deflationists attribute success to true beliefs, but they remain free in principle to handle cases like Callum’s differently.

Second, and more importantly, the falsity of Callum’s belief about the money’s whereabouts or the truth of the corresponding belief in Alison’s case are *not* responsible for the difference with respect to the coincidental/non-coincidental divide between the cases. To see this, consider a variation of Callum’s scenario in which the money is indeed in box X. However, after his choice, the money he wins is not actually what was in the box but rather a donation from the eccentric billionaire, again motivated by Callum's choice of X. We could even stipulate that the money’s placement in the box was a mistake by the show, meaning that without the billionaire’s intervention, Callum’s choice would not have led to the same outcome. However, this stipulation is not strictly necessary. In this modified scenario, the same beliefs that played a role in Alison’s case are still true with respect to Callum’s. Yet, even with the addition of truth to Callum’s explanation of success, the coincidental nature of the outcome remains undispelled, as the following explanation is still unsuccesful:

Callum satisfied his desire to get the money because he *believed truly* that the money was in box X, thus that by choosing X, he would get the money; and his choice caused him to get the money.

If this reasoning is correct, then truth alone cannot be what differentiates these cases. This insight undermines Gamester’s strategy in favor of the non-deflationist: if including truth in Callum’s case does not remove the sense of coincidence, nor can it explain the absence of coincidence in Alison’s case. Both deflationists and their opponents, therefore, should look elsewhere to explain the intuitive sense of coincidence.

Gamester suggests that a meaningful explanatory contrast between the cases requires “something that explains why Alison’s action was successful, but which cannot explain why Callum’s action was so” (2018: 1254). However, if Callum’s belief can indeed be regarded as true while his success remains coincidental, this requirement overlooks an alternative possibility: the distinction might lie in an element present in Callum’s case but absent in Alison’s, rather than the reverse. This alternative offers a straightforward means of differentiating between the two successes, accessible to both deflationists and their opponents: namely, by incorporating the billionaire’s intervention into the explanation of Callum’s outcome:

Callum satisfied his desire for a million pounds because he chose box X & *his choice caused a billionaire to give him a million pounds.*

Accordingly, the difference between Callum’s and Alison’s cases is not due to the truth of one of Alison’s explanatorily relevant beliefs versus the falsity of one of Callum’s. Rather, it stems from the intervention of an unexpected cause—the billionaire’s action—mediating between Callum’s action and his intended goal.

This diagnosis has strong intuitive appeal, and aligns with existing analyses of the notion of a coincidence (see Lando, 2017, with responses by Bhogal, 2019 and De Rizzo, 2021). While differing in specifics, these accounts commonly argue that coincidences are marked by a lack of generalizable patterns or principles that would typically explain the outcome. For example, that two unrelated people look alike or that my hand fits perfectly into a particular concrete imprint (Gamester, 2018: 1252) are considered coincidences partly because the best available explanations address each fact separately, without linking them through a general, explanatorily relevant principle. Similarly, in Callum’s case, there is no underlying principle that connects his choice of box X to the billionaire’s intervention, which crucially determines the outcome. Crucially, this is independent from the truth or falsity of what Callum believes.

### A residual worry (1)

Even if deflationists can ultimately account for the distinction, one might argue that cases like Alison’s still require a general principle linking success-conducive actions to their success, for instance:

*General Success*. For every action A and belief-desire pair B–D explaining it, if A is successful (with respect to D), then A is successful in part *because* B is true. (cf. Gamester, 2018: 1247 for a similar principle)

From the deflationist perspective, truth merely serves as a device for generalizing where ordinary language lacks the capacity. Let us set aside the precise formulation of a “deflated” version of *General Success*—whether as an infinitary conjunction of individual cases, as a principle with quantification over sentences, or some other approach. Admittedly, such reformulations are delicate and might raise specific challenges for deflationism.^8^

Here, however, I would like to focus on a different issue suggested by some of Gamester’s remarks: Can the deflationist capture the explanatory role of *General Success*? Even in Alison’s case, separate from its contrast with the problem scenario, it seems that this principle helps explain why her success is not merely coincidental. Indeed, the aforementioned accounts of non-coincidental outcomes suggest that general principles contribute to the explanatory connection between explanandum and explanans (Lando, 2017; Boghal, 2019; De Rizzo 2021). For example, general thermodynamic principles ensure that the connection between striking a match and its lighting follows a lawful, regulated pattern, rather than being a mere coincidence. The same applies to successful action: Alison’s belief that selecting the box would yield the money is linked to her act of selecting the box in a way that requires a general principle like *General Success*. In some sense, it is partly *because of* General Success that her belief and action are connected.

It remains an open question whether such explanatory roles of laws or principles can be reduced to that of the direct explanans they support—for example, whether we might say, “the match lit because of its striking *and the laws involved*.” Alternatively, it could be that such laws function as higher-order reasons, offering an explanation for the original explanatory connection itself (Skow, 2016). I will leave this question aside for now; it suffices to note that such principles likely play an important explanatory role, either by ensuring non-coincidental connections or by contributing directly to the explanans (see De Rizzo (2021) for discussion).

Deflationary versions of *General Success* may risk losing explanatory power in comparison. The approach of formulating an infinitary conjunction of instances, for example, fails to meet a basic relevance criterion in explanation: it would be irrelevant to cite someone else’s independent belief to explain Alison’s success. Versions using quantification over sentence positions fare better here, as they avoid reference to specific beliefs—but they arguably still leave much to be desired. The most promising deflationary approach might be based on Ramsey’s definition of a true belief:

For every belief B, B is true iff_df_ ∃p (B is a belief that p, and p). (cf. Ramsey, 1927).

Assuming this definition allows substitution on both sides within the scope of “because,” we might arrive at the following deflated version of *General Success*:

General Success*. For every p, For every action A, belief-desire pair B-D explaining A, if A is successful (with respect to D) then A is successful in part *because* ∃p (B is a belief that p, and p).

However, unlike the original *General Success* principle, General Success* does not capture a unified, general pattern linked to a feature of beliefs that lead to successful actions. Beyond being beliefs, there is no substantial commonality between *believing that by nodding, one will get a beer* and *believing that by selecting a box, one will get some money*. *General Success*, by contrast, highlights a common, explanatorily relevant feature shared by successful actions: they are based on *true* beliefs. Only *General Success*, then, can account for the non-coincidental nature that characterizes all instances of successful actions. The explanatory function of *General Success*, therefore, cannot be fully replicated by General Success*, nor is it clear how other deflated versions could do better. (I come back to this objection in Sect. 5.)

## Truth and compositional principles

Another source of discontentment with deflationism stems from the use of the truth-predicate in so-called compositional principles, such as:

“p & q” is true iff “p” is true and “q” is true

“p ∨ q” is true iff “p” is true or “q” is true

As noted earlier, it is desirable for a theory of truth to include such principles alongside instances of the T-schema. But how should a deflationist interpret these compositional schemes? To render them as clear, assertible principles, we must express them as generalizations. For instance (where “S” and “T” are first-order variables):

*&-Comp*. For all sentences S and T, ⌜S & T⌝ is true iff S is true and T is true

The central question, then, is on what basis deflationists can justify *&-Comp* and similar compositional principles.

To examine this, consider the limitations of attempting to derive compositional principles solely from the T-schema, focusing on the case of conjunction (following Heck, 2021):

P1. “p & q” is true iff (p and q)

P2. “p” is true iff

P3. “q” is true iff q

We also assume a substitution rule to aid us:

(Sub) *Substitution of equivalents*: φ(p), p iff q ⊢ φ(p/q)

(See Larson & Segal, 1995: 28; ‘Replacement’ in Lepore & Ludwig, 2007: 32).

Then P1–P3 give us rather straightforwardly

C. “p & q” is true iff “p” is true and “q” is true

Notice that, if we understand this as an argument schema, then it gives us a recipe to prove every instance of *&-Comp*. But it falls short of proving *&-Comp* itself as a general principle (Gupta, 1993; Shapiro, 1998). If, on the other hand, we interpret P1-P3 as first-order generalizations over sentences, we would also need a rule for universal elimination, which leads to a new issue: “p” and “q” would then function as variables appearing both inside and outside quotation marks. This complication already arises for deflationists with respect to the T-schema itself.

In response, a deflationist might adopt substitutional quantification throughout. Yet, if we formulate C in this substitutional manner, it arguably still fails to capture *&-Comp* fully (Gupta, 1993; Heck, 2021). The substitutional form of C would apply only to specific instances in a given language. However, *&-Comp* entails a broader commitment: not only must it hold for current instances, but it should also apply to all extensions of the language (perhaps even necessarily). Since one would quantify both inside and outside quotation marks, sui generis higher-order quantification in the position of sentences would not by itself resolve the issue without changing the premises considerably (one would have, for instance, to mimic the relation between “p” and p by other means, for instance a meaning relation or similar).

### Field’s strategy

Field (2006) acknowledges the former challenge and provides a way for the deflationist to meet it. In a nutshell, his proposal is to include directly rules in the metalanguage that enables one to pass from a scheme that we’ve shown to hold with respect to schematic letters “p_1_”,… “p_n_”, to the corresponding generalization, e.g.:

(…) if θ(‘p_1_’,…,’p_n_’) is a schema in which all occurrences of the schematic letters p_1_, …, p_n_ are directly surrounded by quotes and not in the scope of further quotes, then if one has inferred

θ(‘p_1_’,…,’p_n_’)

one is licensed to conclude

For any sentences x_1_,…, x_n_, θ(x_1_,…,x_n_). (2006: 14)

To illustrate this, once we’ve proven that

C. “p & q” is true iff (“p” is true and “q” is true)

where “p” and “q” are schematic letters in the metalanguage, we might apply the proposed rule to obtain

For every sentence S, for every sentence T: ⌜S & T⌝ is true iff (S is true and T is true).

Which is *&-Comp*.

### The logical objection: Heck’s overgeneralization argument

In a recent response, Heck (2021) argues that Field’s approach overgenerates compositional principles, in that it produces analogous principles even for non-truth-functional connectives. According to Heck, this overgeneration indicates that “what Field’s method yields simply are not compositional principles as they are understood in semantic theory” (121).

Before examining Heck’s specific examples, a preliminary remark is needed. Deflationists are committed to the idea that T-biconditionals express more than mere material equivalence. For example, deflationists maintain that the disquotational nature of truth helps explain sentences like:

The axioms of Euclidean geometry are not all true, but they might have been.

As Heck notes, the above example is not true “for the boring reason that the sentences that express the axioms of Euclidean geometry might have meant something else” (118). Therefore, T-biconditionals must be at least as strong as intensional equivalences. Thus, the deflationist should not only endorse:

“p” is true ↔ p.

But also:

Necessarily, (“p” is true ↔ p)

(Where the double arrow expresses material equivalence.)

This has surprising consequences, particularly for counterfactuals, as illustrated by Field (1994):

Even if “snow” had meant grass, “snow is white” would still have been true.

Interpreting this as true seems strange, in part because plausibly a counterfactual change in a word’s meaning should affect the truth-value of a sentence containing it. For the deflationist, this is not an issue: as Field explains, by accepting intensional equivalences as definitional of truth, the deflationist effectively “fixes” the meanings of sentences as they are in the actual world. This is a price the deflationist is willing to pay to capture generalizations involving truth, including those within the scope of modal operators.

The deflationist’s reliance on T-biconditionals, however, goes even further. For instance, they want to capture statements like:

It is sometimes possible to see objects behind the sun *because* the axioms of Euclidean geometry are not all true.

As Heck nicely puts it, this “is supposed to be a way for me to affirm that the non-Euclidean character of space is responsible for the somewhat surprising behavior of photons, not to make the absurd claim that optics is beholden to the semantics of English. But if that is to be so,” he continues, “then “The axioms of Euclidean geometry are all true” must simply express what the axioms of Euclidean geometry jointly do and, in particular, must not have any extra, ‘semantic’ content.”(118–119) Given the parallel with the modal case, the lesson for deflationists is that they should permit the right- and left-hand sides of T-biconditionals to be intersubstitutable within the context of hyperintensional connectives like “because.” This, Heck argues, is an implication of the deflationist’s approach: if the truth predicate is merely disquotational, then “p” and “p” is true” are equivalent in a suitable strong sense that underlies those substitutions (Heck, 2021: 119–121; Heck highlights that this commitment also follows from the standard response to the success objection considered before).

Let us turn now to Heck’s cases that illustrate how Field’s strategy overgenerates compositional principles. Heck illustrates first the overgeneration with the derivation of a compositional principle for “because”:

P1. “p because q” is true iff (p because q)

P2. “p” is true iff p

P3. “q” is true iff q

C1. “p because q” is true iff (“p” is true because “q” is true).

C2. *Bec-Comp*. For all sentences S and T, ⌜S because T⌝ is true iff (S is true because T is true).

And a few pages later, also with the necessity operator:

P1. “Necessarily, p” is true iff necessarily, p

P2. “p” is true iff p

C1. “Necessarily, p” is true iff necessarily, “p” is true

C2. *Nec-Comp*. For every sentence S, ⌜Necessarily, S⌝ is true iff necessarily, S is true.

What is problematic about these compositional principles? First, note that while one might correctly object to the use of material conditionals only to derive C2 in each case, as we noted before, the deflationist is independently committed to a stronger interpretation of these equivalences, one that supports these derivations. Although Heck raises potential concerns about how exactly to specify this increased strength with respect to “because” (I will address these issues shortly), he does not consider these derivations flawed in themselves. Rather, it is the very fact that such derivations are possible that casts doubt on Field’s proposal, even in the simpler *&-Comp* case. Heck’s own words are instructive here (I quote him at length):

Friends of semantics will have greeted

[Bec-Comp.] For all sentences S and T, ⌜S because T⌝ is true iff S is true because T is true.

with bemusement. But what exactly is supposed to be wrong with it? Why does it feel so different from

[&-Comp.] For all sentences S and T, ⌜S & T⌝ is true iff S is true and T is true

And related principles? As [&-Comp.] is generally understood by friends of semantics, what it says, in part, is that the truth-value of a conjunction is entirely determined by the truth-values of its parts, i.e., that conjunction is truth-functional. It is the truth-value of the conjuncts that matters, but what the conjuncts contribute is just their truth-value and nothing else. Similarly, then, [Bec-Comp.], as understood by friends of semantics, would tell us that the truth-value of “A because B” is wholly determined by the truth-values of A and B and the causal relationship between those truth-values (e.g., by whether the True because the False). That is barely coherent, but never mind. What the provability of [Bec-Comp.] really shows us is that the proof of [&-Comp.] goes through whether or not “and” is truth-functional. So, if [&-Comp.] is understood in the sense in which it is schematically provable, it does not affirm the truth-functionality of conjunction; so understood, then, it is not the compositional principle for “and” as friends of semantics would understand it; that principle is not schematically provable*.* (132; square brackets adjust to our terminology)

In short: the fact that we can prove *Bec-Comp* is supposed to show that *&-Comp*, when established by purportedly the same means, fails to capture the truth-functionality of conjunction, which is integral to the usual practice of semantics. Heck takes this to show “that the notion of truth, as it appears in semantics, is not playing the merely expressive role that a disquotational truth predicate plays: One cannot understand the use of the truth-predicate, *in semantics*, as purely disquotational, as just a tool of ‘semantic ascent’. Rather, the notion of truth is doing serious theoretical work, and that work is nowhere more visible than in disputes over truth-functionality.” (133).

### A response

Heck raises several intriguing issues in the quote above. Before addressing them, it’s helpful to clarify a few points.

First, a point of consensus: the connective “because” is not truth-functional, meaning that the truth-value of a sentence like “p because q” is not fully determined by the truth-values of its components “p” and “q.” It’s well-known that even necessarily equivalent sentences cannot always be substituted within the scope of “because” without affecting the truth of the sentence. For instance, “Someone is Greek because Socrates is” is true, but “Someone is Greek because Socrates is & 2 + 2 = 4” is not. Hence, “because” is hyperintensional. (I follow Heck in assuming that “&” (i.e., “and”) is truth-functional.)

I also agree with Heck’s interpretation of truth-functionality itself: in the case of conjunction, the truth-value of the whole conjunction is *wholly determined* by the truth-values of its sentence-parts. However, it’s doubtful that this idea of “determination” is adequately captured by the material equivalence expressed in *&-Comp*, the usual understanding by friends of semantics notwithstanding (Schnieder, 2008). In itself, *&-Comp* states only a generalized material equivalence between instances of ““p&q” is true” and ““p” is true and “q” is true.” But material equivalence is symmetric, whereas the relevant sense of determination is not.

For present purposes, however, we might acknowledge, with Heck, that *&-Comp* is generally interpreted as conveying the truth-functionality of conjunction, whatever closer scrutiny might reveal about this interpretation. Part of this interpretation assumes that the truth-value of the complex sentence in question is “*entirely* determined” by the truth-values of its components.

However, as Heck himself notes, *Bec-Comp* doesn’t amount to this. Heck reads *Bec-Comp* as indicating that “the truth-value of ‘A because B’ is wholly determined by the truth-values of A and B *and the causal relationship between those truth-values*” (emphasis added). Thus, *Bec-Comp* does *not* affirm that “because” is truth-functional. Consequently, Heck’s criticism is not that, since a schematic proof can apply to both cases, either both “and” and “because” are truth-functional or neither is; his critique isn’t that deflationists fail to capture a distinction in truth-functionality between the two cases.

On the other hand, a crucial difference between the proofs of *&-Comp* and *Bec-Comp* lies in the strength of the substitution rules and the corresponding equivalences (the “iff”s in P2–3). Therefore, Heck’s charge cannot rest on the *same proof* being extended from *&-Comp* to *Bec-Comp*, or vice-versa. Surely, the stronger substitution rule required for *Bec-Comp* plausibly subsumes the weaker, material equivalence substitution required for *&-Comp*. Strictly speaking, however, the means required for the proofs of each principle remain importantly different, even if the proofs qualify as schematic in Field’s sense. Such a difference in required means should be taken into account if proofs have any chance of bearing on the sense of what they establish, as Heck seems to suppose. It remains unclear why the mere provability of *Bec-Comp* by alternative means should cast doubt on whether *&-Comp* adequately captures the truth-functionality of conjunction.

Perhaps the commitment to *Bec-Comp* itself is problematic, irrespectively of its consequences for *&-Comp*. While Heck does not dwell on it, he hints at a potential issue when he writes:

[Bec-Comp], as understood by friends of semantics, would tell us that the truth-value of “A because B” is wholly determined by the truth-values of A and B and the causal relationship between those truth-values (e.g., by whether the True because the False). *That is barely coherent*, but never mind. (2021: 132, my italics)

Let’s assume, then, that the notion of “determination” has indeed been read into the material equivalence of *Bec-Comp*. On this reading, *Bec-Comp* states that, in addition to the truth-values of the component sentences, the truth-value of “p because q” depends on whether it is true that “p” is true because “q” is true. But is this condition incoherent, or otherwise problematic for deflationism?

According to Heck's reading, this condition would imply a causal relationship between truth-values—a notion that is, indeed, difficult to make sense of. However, this is not actually what *Bec-Comp* says. First, “because” need not express a causal relationship; there are non-causal explanations (Correia & Schnieder, 2012; Kim, 1974). Moreover, nothing in *Bec-Comp* implies that explanatory relationships must exist *between* truth-values, whether causal or otherwise, for any “because” claim to hold true. This point also applies to truth-functional connectives. Even if we consider truth-values as the *referents* of sentences, we don’t have to interpret *&-Comp* as implying that the truth of “p&q” depends on the existence of a “conjunctive relationship” between the True and itself. Any semantics that treats “because” as asymmetric and factive must go beyond assigning mere truth-values as the semantic values of sentences it relates.^9^ Once finer-grained distinctions are permitted, one ought to distinguish determination by how *truth attributions* relate—specifically, that “p & q” is true because “p” is true and “q” is true—from determination by whether *truth-values* themselves relate that way. The former interpretation is not only intelligible and unproblematic but also better aligned with what *Bec-Comp* actually entails.

### Further issues

Even if a deflationist might thus accept *Bec-Comp*, aren’t there further cases that show that Field’s strategy is overly prolific in generating bad compositional principles? Field (2006) himself acknowledges concerns about extending this approach to belief contexts. Consider a compositional principle involving a sentential operator like "Galileo believes that." A proof for such a principle might look like this:

P1. “Galileo believes that p” is true iff Galileo believes that p

P2. “p” is true iff p

C1. “Galileo believes that p” is true iff Galileo believes that “p” is true

C2. For all sentences S and T, ⌜Galileo believes that S⌝ is true iff Galileo believes that S is true

It’s well-known that C1 has counterexamples, and thus C2 is false: for instance, Galileo may not have spoken English, or he may have had idiosyncratic beliefs about truth ascriptions. These counterexamples illustrate that even if one accepts T-equivalences in a sufficiently robust deflationist framework—one that accounts for cases involving “because” and “necessarily”—C1 remains problematic (Field 2006: 23). As Field notes, this method should not extend to belief or other propositional attitudes.

The most straightforward way to block this derivation is to limit the substitution of equivalents to material equivalence only, as Field (2006: 23) seems to propose. Specifically, substitution of equivalents shouldn’t apply within the scope of belief operators (like "Galileo believes that"). However, as Heck points out, such a restriction would also prevent deriving *Bec-Comp* and *Nec-Comp*, which rely on a stronger substitution rule. This is problematic for deflationists, as the disquotational character of truth requires a substitution rule that applies within the scope of “because” and “necessarily”, which commits them to *Bec-Comp* and *Nec-Comp*.

But there is room for a deflationist response. Much of the discussion revolves around finding the “right” strength of equivalence in T-biconditionals that allows substitution in the scope of certain operators, like “necessarily” and “because.” With an appropriately defined equivalence, deflationists could formulate a substitution rule that supports the derivation of compositional principles for these operators without overextending to propositional attitudes. One way to define such an equivalence is through the notion of *because-equivalence* (symbolized by “⇔”), schematically definable thus:

p ⇔ q iff_df_ ((p because r) iff (q because r)) (cf. Krämer, 2019; Correia, 2016)

Accordingly, what “p” and “q” express are explanatorily equivalent when they share the same reasons why they hold; importantly, for simplicity, the notion of “because” is *non-factive* in the definition: it might be that *p* because *q* even if it is not the case that *p* nor that *q* (Fine, 2012: 48–50). With *because-equivalence* in place, the strengthened T-biconditionals take the form:

□(‘p’ is true ⇔ p)

(Recall that Field already considered the strengthening by necessarily equivalence.)

This new T-schema is stronger than both material and intensional equivalence. Accordingly, the disquotational nature of truth would not only preserve truth and necessary truth but also the *reasons* for truth: necessarily, whatever explains why ‘p’ is true also explains why p. The substitution rule would then be

φ(p), □(p ⇔ q) ⊢ φ(p/q)

This approach supports the derivations of *Bec-Comp* and *Nec-Comp* without overextending to belief operators. Does *because-equivalence* entail substitutivity within the scope of propositional attitudes? Plausibly, no. For instance, Galileo may still have beliefs about truth ascriptions that conflict with the stronger T-biconditionals, just as he might with the weaker ones.

One is right to point out that this approach is tailored to work for “necessarily” and “because”, and that it might just be a contingency of the examples considered that they are formulated in terms of these operators. But the principled conjecture the deflationist might offer is that just as necessary equivalence provides the right amount of individuation or grain to support substitution across contexts generated by non-representational *intensional* operators throughout, such as “it is necessary that”, “it is possible that”, and so on, *because-equivalence* provides the right amount of individuation to support substitution across contexts generated by non-representational *hyperintensional* operators as well, such as “it is essential to… that…” and “it is a law that”. (By “non-representational,” I mean operators that are not sensitive to the mode of presentation of content, thereby excluding propositional attitudes and certain hyperintensional operators like “means that.”).

### A residual worry (2)

The deflationary theory of truth, once augmented by strengthened T-biconditionals, offers a promising approach for handling cases involving terms like “because” and “necessarily” without extending to propositional attitudes (assuming the conjecture above). However, it faces a widely discussed challenge: its inability to account for the dependence of truth on reality (cf. Newman, 2002: 34ff., Vision, 2005; for responses, see McGrath, 2003 and Thomas, 2011). Just as the *success objection* highlights the explanatory power of truth ascriptions, part of our truth-related practice involves explaining those ascriptions by reference to the actual state of affairs they describe. Thus, if the sentence “Snow is white” is true, it is true *because* snow is white.^10^

The deflationist who accepts strengthened T-biconditionals, however, cannot endorse this explanatory link. According to the strengthened T-biconditionals, any explanation for why “Snow is white” is true would, in effect, be an explanation for why snow is white, since the equivalence expressed in □(“Snow is white” is true ⇔ Snow is white) would make them intersubstitutable in contexts of explanation. If we claim that “Snow is white” is true because snow is white, it would follow that snow is white because snow is white. This is false, as “because” in its intended, objective explanatory use is irreflexive—there is no self-explanation.^11^

In fact, the problem of accounting for truth’s dependence on reality arguably becomes more pressing with the strengthened T-biconditionals than it was with the original material ones. The issue with the original material biconditional is often that it may simply lack the strength needed to capture the asymmetry of truth’s dependence on reality. With the strengthened biconditionals, however, the objection is that the theory actively misrepresents the nature of truth-dependence: by its terms, it outright *denies* that “Snow is white” is true *because* snow is white. The strengthened theory, then, takes a stance—though it is the wrong one from the objector’s perspective.

## Aristotelian Deflationism

In the *Metaphysics* and the *Categories*, Aristotle writes:

It is not because of our having the true thought that you are pale, that you are pale; rather it is because of your being pale that we who say so have a true thought. (Met. θ 10: 1051^b^ 6–9)

It is because of the thing’s being, or not being, thus-and-so that the predication is said to be true or false.. (Cat. 5: 4^b^8–10)

By interpreting “thought” in terms of belief, the first quote suggests the following principle:

1. (Necessarily:) If x believes truly that p, then (x believes truly that p) *because* p.

By interpreting “predications” as sentences, the second quote suggests the following principle:

2. (Necessarily:) If “p” is true, then “p” is true *because* p.

The second principle—or something close to it—is sometimes called “Aristotle's insight on truth” (see Künne, 2003: 150; Correia & Schnieder, 2012: 26; see Bolzano, 1837: Sect. 205). We can set aside the (tedious) question of whether one principle reduces to the other; they are naturally seen as complementary, and we will treat them as such. Though Aristotle himself doesn’t explicitly address this, it is plausible to think that if these principles hold, then their modally strengthened versions hold as well, since these principles can be understood as constitutive of the concept of truth and of truth attributions to propositional attitudes. Let us refer to these modally strengthened principles—those prefixed with “necessarily”—as *Aristotle’s Schemata*. Aristotle's Schemata embody the core idea behind the slogan that truth, in thought or discourse, depends on reality, which underpins the concern raised at the close of the last section.^12^

Now, what if an open-minded deflationist were to adopt Aristotle’s Schemata? I will conclude by discussing the implications of this choice for both the issues examined earlier and the broader questions they raise. By embracing an Aristotelian approach, the deflationist could temper their assumptions with respect to the success and the logical objections, while addressing both of the previously raised residual worries:

*Worry 1*. That the deflationist cannot account for the explanatory role of general principles linking succcesful behaviour to true beliefs (e.g. *General Success* above).

*Worry 2*. That the deflationist cannot account for truth’s dependence on reality (e.g. that “snow is white” is true because snow is white).

### The success objection revisited

Non-deflationists who advance the success objection typically hold that statements (3) and (4) below are not equivalent:

3. Elisa satisfied her desire for a beer *because* Elisa believed that by nodding she would get a beer; and by nodding, she got a beer.

4. Elisa satisfied her desire for a beer *because* Elisa believed *truly* that by nodding she would get a beer.

As noted earlier, a deflationist might respond by adopting the following principle:

For every belief B, B is true iff ∃p (B is a belief that p, and p).

Or equivalently (and better suited for the present context):

For every x, x believes truly that p iff x believes that p and p. (cf. Ramsey, 1927).

One could argue that (3) and (4) are equivalent by interpreting this definition as entailing that the statements “Elisa truly believed that by nodding, she would get a beer” and “Elisa believed that by nodding, she would get a beer, and by nodding, she got a beer” are interchangeable *salva veritate* in the context of “because.” Since this reasoning generalizes, it suggests that explanations of type (3) and type (4) do not differ in extension.

But, instead of Ramsey’s definition, consider the following principle, which aligns with Aristotle’s schemata:

*Bel-Bec*. For every x (x believes truly that p *because* (x believes that p, and p)).

(Notice that *Bel-Bec* goes only beyond Aristotle’s first schemata in including “x believes that p” in the explanans.) This principle yields an interesting result: any explanation given in terms of true belief entails a corresponding explanation that does *not* rely on truth. Thus, from

4. Elisa satisfied her desire for a beer *because* Elisa believed *truly* that by nodding she would get a beer.

Together with the immediate consequence of an instantiation of Aristotle’s schema

Elisa believed *truly* that by nodding she would get a beer because she believed that by nodding she would get a beer, and by nodding she would get a beer.

And the transitivity of “because”, one gets the truth-free explanation

Elisa satisfied her desire for a beer *because* she believed that by nodding she would get a beer, and by nodding she would get a beer.

In contrast to the conservative deflationist, who seeks to show that truth can be “eliminated” from explanations, the Aristotelian approach show that truth’s explanatory role is dispensible. Truth ascriptions, being always explainable, are in this sense never essential to explanations; what truth explains can always be accounted for without invoking truth itself.

This result, while weaker than the more conservative deflationist stance, might suffice. Just as a physicalist who believes that every phenomenal fact is explained by physical facts might still allow that phenomenal facts can explain without undermining their view—as long as an alternative physical explanation is available for every explanandum (Kroedel & Schulz, 2016)—so too might an Aristotelian deflationist acknowledge that truth has an explanatory role, though not an indispensable one. By the lights of Aristotle’s schemata, each truth-based explanation can always be accompanied by an alternative explanation independent of truth terms.

The first residual worry—that the deflationist cannot account for the explanatory role of general principles connecting successful actions and true beliefs—can be met by the Aristotelian. The crucial point is that, unlike the more conservative deflationist, the Aristotelian is comfortable allowing truth, and true beliefs, to play an explanatory role. Accordingly, they are open to endorsing principles in the spirit of: “Successful actions are succesful in part because true beliefs prompted them.” The issue is not that truth cannot figure in explanations at all, but rather that any explanatory role it plays can, at a more fundamental level, be traced to disquoted instances. To illustrate, consider again the principle:

*General Success*. For every action A and belief-desire pair B-D explaining it, if A is successful (with respect to D), then A is successful in part *because* B is true.

*General Success* is a universal generalization, which holds because its true instances do (Fine, 2012). Take, for example, the following instance:

If Alison’s choice of box X is successful, then it is successful in part because Alison’s belief that by choosing X, she would get the money, is true.

Since the antecedent holds, then:

Alison’s choice of box X is successful because Alison’s belief that by choosing X, she would get the money, is true.

Given that true conditionals are explained by either the failure of their antecedents or their true consequents (Fine, 2012; Schnieder, 2011), the latter statement is a reason why the former holds. But Alison’s belief is true, according to the Aristotelian, because by choosing X, Alison would get the money. Thus by transitivity of “because”, we get:

Alison’s choice of box X is successful because by choosing X, Alison would get the money.

Further applications of transitivity let us reach an explanation of *General Success* without appeal to truth. For the Aristotelian deflationist, the principle “Successful actions are successful in part because true beliefs prompted them*”* need not be reformulated to exclude mention of truth. Rather, the Aristotelian is satisfied that any explanatory force the principle appears to have can ultimately be traced to more basic explanations—explanations that do not themselves invoke truth.

### The logical objection revisited

Similarly, a possible approach to handling “because” claims, such as those involving (non-)Euclidean geometry as pointed out by Heck, is to adhere to stronger T-biconditionals (Heck, 2021: 118–121). However, an interesting alternative is offered by the Aristotelian scheme. Take again the following claim:

1. It is sometimes possible to see objects behind the sun *because* the axioms of Euclidean geometry are not all true.

For simplicity, we might consider instead

1*. It is sometimes possible to see objects behind the sun *because* the axioms of hyperbolic geometry are all true.

(Assuming, for the sake of argument, that hyperbolic geometry accurately describes space.)

Let “Δ” be the plurality of sentences expressing the axioms of hyperbolic geometry. Then 1* can be reformulated as:

1*. It is sometimes possible to see objects behind the sun *because* “Δ” are all true.

From 1*, the instantiation of the scheme

2. (Necessarily) If “p” is true, then “p” is true *because* p.

(with the obvious extension to pluralities), and the transitivity of “because”, we arrive at

3. It is sometimes possible to see objects behind the sun because Δ.

This formulation provides an explanation without the notion of truth, sidestepping the need for hyperintensional equivalence between “p” and ‘‘“p” is true”.

Similarly, as Heck observes, to capture the proper reading of the sentence

The axioms of Euclidean geometry are not all true, but they might have been*,*

it must be required that, necessarily, if the axioms are true, then what they state holds. That is, letting “p” be the conjunction of the sentences expressing the axioms of Euclidean geometry,^13^ the following must hold:

Necessarily (“p” is true → p).

The relevant instance of the Aristotelian Schemata here is:

Necessarily (“p” is true → (“p” is true because p)).

(Recall that the deflationist is ready to counter the objection that turns on "p" possibly varying in meaning (see §3.2 above); I assume the Aristotelian might follow suit.) But since “because” is factive in this context, this instance entails that necessarily, (“p” is true → p). Hence, the modal cases can also be accommodated within the proposed Aristotelian framework.

These considerations demonstrate that the Aristotelian Schemata free us from relying on robust substitution rules to interpret the embedding of truth ascriptions within modal operators and “because” contexts. Thus neither *Bec-Comp* nor *Nec-Comp* are derivable from the Aristotelian Schemata, even if Field’s proposed procedure is adopted (since the rule with material equivalence is reasonably weak, and is an integral part of truth theories, *&-Comp* can be readily accounted for). This becomes evident when we examine the premises of the derivation for *Bec-Comp* as adapted to the present context:

P1. “p because q” is true because (p because q)

P2. “p” is true because p

P3. “q” is true because q

?C1. “p because q” is true iff “p” is true because “q” is true.

C1 does *not* follow from P1–P3, nor does the analogous claim obtained by replacing “iff” with “because”. While additional assumptions might allow one to derive *Bec-Comp* and *Nec-Comp*, the preceding discussion only established that these principles are *acceptable* to the deflationist—not that they are *desirable*. In this respect, the flexibility of the Aristotelian approach is a strength, allowing one to refrain from commitment to such principles without inconsistency.

Furthermore, the Aristotelian account restores the consistency of deflationism with a dependency of truth on reality, central to the second residual worry. Indeed, incorporating the Aristotelian Schemata is arguably the most straightforward way to integrate this dependency.

More often than not, “arguably” is a signpost for trouble. Indeed, many authors would challenge the claim made in the previous paragraph. Accordingly, the slogan *“truth depends on being”* requires a robust ontology of *truth-makers*—such as facts, tropes, or the like—that serve as anchors for truth. The *reasons why* provided by the Aristotelian Schemata are insufficient for capturing the worldly dimension of this alleged dependency. A proper explanation would need to explicitly reference, for example, *the fact that snow is white* or *the snow’s whiteness*.

The debate is complex, and I cannot fully address it here. However, it is worth noting that the right-hand side of instances of Aristotelian Schemata can be as much about the world as any statement is. For instance, “Snow is white” is true because *snow is white.* The latter claim is about snow and its color. It just so happens that to state it, we simply unquote the sentence whose truth is being explained, but that is surely not essential to the strategy (the meta-language need not be English), and should not obscure the fact that we are pointing to bits of the world in explaining why the sentence is true.^14^

Moreover, the requirement that the being upon which truth depends must be reflected in (first-order) ontological commitments stands in need of substantial defense—just as the supposed superiority of e.g. “Snow is white” is true because the fact that snow is white obtains” or “Snow is white” is true in virtue of the fact that snow is white” over the Aristotelian alternatives does. Even if this requirement is ultimately justified, the Aristotelian can simply concede the point and adopt truthmaker-friendly variants such as: “(Necessarily), if “p” is true, then “p” is true because the fact that p obtains.” Yet in light of the plausible claim that whenever p, then *the fact that p obtains because p*, the Aristotelian Schemata resurface—now refracted through the lens of the truthmakers some insist upon (cf. Schnieder, 2006). The same explanatory consequences outlined in this section would then continue to apply.^15^

Finally, regardless of whether the latter strategy is adopted, it remains debatable whether the approach just outlined truly merits the label “*deflationist*.” Strict deflationists may reasonably resist assigning even minimal explanatory roles to truth, or may be uneasy with invoking a hyperintensional connective like “because.”^16^ While the position attempts to strike a middle ground between conservative deflationism and more robust theories of truth, it leans toward the deflationist side (at least so long as it refrains from invoking truth-makers). If the deflationist in the style of Field is willing to endorse necessitated truth-schemata, then the proposed strengthening in terms of “because” appears to be a natural next step. Admittedly, the label *“deflationism”* draws attention to the negative core of the Aristotelian position—not the *eliminability* of truth, as orthodox deflationists might insist, but rather its *explanatory dispensability*. Insofar as non-deflationists typically assign an indispensable explanatory role to truth-ascriptions, the label is not without justification. Yet if substantivity does not entail explanatory indispensability, then there is room to argue that the Aristotelian view allows for a substantive conception of truth. In that light, the title *“deflationism”* should perhaps be taken with a pinch of salt. With or without the scare quotes, Aristotelian “deflationism” warrants closer scrutiny, especially given its promise in addressing the challenges outlined above.

## Conclusion

True beliefs play a role in facilitating and explaining successful behavior. Deflationists offer alternative explanations that avoid direct reference to truth, focusing instead on beliefs and what they represent. Gamester (2018) argued that this approach fails to distinguish between coincidental and non-coincidental success, as illustrated in Callum’s case. However, the deflationist can account for this distinction if they allow explanations to extend beyond just beliefs and their contents—an allowance that is reasonable, as truth alone does not draw this distinction.

Compositional principles connecting truth to complex sentences are fundamental to our understanding of truth. Deflationists might follow Field (2006) by introducing generalization rules that enable these compositional principles. Heck’s concerns rest on overstating the similarity of proofs compositional principles for truth-functional connectives, such as conjunction, and that of principles relating to non-truth-functional connectives, such as necessity and explanation, underwritten by Field’s proposal. Further overgeneralization worries can also be addressed, provided the T-schema relies on a hyperintensional notion of equivalence couched in terms of “because”.

Despite these responses, some dissatisfaction remains. As Gamester notes, it remains unclear how deflationists can capture the role of generalized principles linking true beliefs to successful actions. Additionally, by introducing hyperintensional equivalences, deflationists risk obscuring the dependence of truth-ascriptions on what true sentences state. But an alternative route is available: the T-schema might be reframed in terms of necessity and “because,” an approach inspired by Aristotle. *Aristotelian deflationism* not only responds to the concerns raised by Gamester and Heck but also addresses the lingering issues. While this may not establish its superiority, it nonetheless demonstrates that *Aristotelian Deflationism*—a perspective largely overlooked in the truth literature—warrants further exploration.

## Footnotes

1 Modulo the paradoxes (Horwich, 1998: 40–43). I leave the restriction aside.

2 I leave open whether the fact that truth serves these uses follows from the disquotational characterization, or whether it is an additional commitment. See Gupta (1993): 62ff. for discussion.

3 Terminology is far from unified in the literature (cf. Künne, 2003: 18), and doubtlessly there are views under the label “deflationism” which would not fit this common characterization. Though I will focus on deflationist views as characterized, much of what will be said applies to closely related views, for instance, which apply the truth predicate not (only) to sentences, but to propositions (e.g. Horwich, 1998), beliefs, and other candidates for truth-bearers. I choose sentences primarily to align with the literature I’m engaging with on what I call the “logical objection”, which treats sentences as truth-bearers. Though compositional principles in semantics certainly rely on ascriptions of truth to sentences, the discussion could be cast in terms of other truth-bearers, provided they have a corresponding internal structure. I thank an anoynmous referee for suggesting to be explicit about this.

4 A related line of criticism centers on compositional principles relating reference and satisfaction to truth. I leave that aside here. See Leeds (1978), Putnam (1978).

5 The objection becomes more pressing given that compositional principles may offer the strongest justification of reflection principles, central to proof theory. Specifically, for any formula ϕ in a suitable arithmetical language, if ϕ is provable, then ϕ itself must hold. For further discussion, see Ketland (1999) and Tennant’s response (2002). For a comprehensive overview of the debate, along with a novel contribution, see Horsten and Leigh (2016).

6 *Transitivity*. If (p because q&r) and (q because s&t), then (p because ((s&t)&r)). Notice that “because” may indicate only partial explanations. I assume that (p because q&r) entails (p because q) (where the latter expresses at least a partial explanation). While transivity is not usually formulated in these terms—more often than not “because”, or a corresponding operator of ground, is allowed to apply to a plurality of sentences stating the grounds in place of the conjunctions above—it is generally accepted in the literature on explanation that “because” should support some form of transitivity, which allows for the common practice of chaining explanations (Fine, 2012; Schnieder, 2011). For dissenting views on transitivity in causal contexts, see Ehring (1987); for non-causal contexts, see Schaffer (2012). Notably, transitivity is not a primary point of contention between deflationists and their opponents in this section.

7 The claims referenced between brackets are distinct—Gamester in fact refers to premises and conclusions in arguments of the form suggested by Horwich (1998): 22–23 on the deflationist’s behalf. The difference is insubstantial in the present context, given that the explanatory claims we use incorporate the main steps in those arguments. Notably, the belief on the money’s location is absent from either. However, as discussed in the next section, including it would not make a substantive difference, since Gamester’s point centers on the belief’s truth in Alison’s case, not the belief per se.

8 See Gupta (1993); on whether sentential or propositional quantification is acceptable by deflationist’s lights, see Horwich (1998): 25ff. for skepticism, and Hill (2001), Båve (2013), Wrenn (2023) for defenses.

9 Being a connective, “because” need not express a relation. I use “relates” in an extended sense here for the purpose of discussion.

10 The challenge does not depend on sentences as truth-bearers: the same intuition would lead us to say that (*it is true that* p) because p, or that the proposition that p is true because p. Indeed, Aristotle’s insight on truth stated below is usually couched in terms of propositions. See the references below. I thank an anonymous referee for pressing me to be explicit about this.

11 As an anonymous referee rightly observes, the concern arises only if we assume that “because” in *because-equivalence* is used in the same sense as in statements like “ “Snow is white” is true because snow is white.” As noted above, the relevant reading of “because” is restricted to its objective, explanatory use—where, in a statement of the form “p because q,” the clause “q” provides a *reason* *why* p is the case. It is difficult to see how one could plausibly depart from this reading in either of the relevant cases, particularly if *because-equivalence* is intended to capture examples such as those discussed by Heck (e.g., concerning Non-Euclidean geometry), and if the dependence of truth on being is to be understood in explanatory terms. That said, even within the objective use of “because,” it is possible that different cases call for distinct ways of individuating explananda and explanantia—a valuable point raised by the referee. But even if that turns out to be true (which is far from obvious), the explanatory chains that lead to failures of irreflexivity remain compelling, unless one arbitrarily treats these uses as distinct solely on the grounds of such fine-grained differences in the relata of explanation. I thank the referee for discussion.

12 As an anonymous referee notes, the dependence of truth on being underpins approaches that use the original T-schemas in terms of material equivalence. Tarski himself explicitly states that the T-schemas are intended to rigorously express Aristotle’s dictum on truth's dependence. However, as mentioned above, it is widely agreed that biconditionals, being symmetric, fail to capture the asymmetry inherent in this dependence. While it may be possible to restore this asymmetry through some feature that distinguishes between the meta- and object-languages, the key point is that the biconditionals alone are insufficient. The use of the objective “because” is proposed as a way to address this shortcoming. For further discussion, see Künne (2003: Sects. 3.5 and 4.1).

13 Since the usual axiomatization by Tarski has the axiom schema of continuity, the conjunction would not be of finite length. I ignore this detail at present.

14 Nominalizations are ubiquitous in English and might simply reflect an expressive convenience, not a “joint in reality”. We might thus allow that in some sense the Aristotelian does point to a fact or the like in endorsing instances of the Schemata, albeit not explicitly, by means of a referring term, in the context of the schema. I assume that the objector relies on a more substantive conception of facts (or whatever serves the role of truth- and falsity-making).

15 I thank an anonymous referee for raising the objection discussed in these paragraphs.

16 A deflationist treatment of “because” in truth-conditional semantics is forthcoming if one allows for rules of inference, alongside axioms, to specify meanings. This is suggested, though not explored further in Williamson (1984), Field (2006).
